# Extending participant feedback beyond clinical studies: A modular system designed to connect researchers and participants

**DOI:** 10.1017/cts.2025.10184

**Published:** 2025-11-03

**Authors:** Alicia Giordimaina Carmichael, Donna Walter, Brandon Patric Labbree, Boluwatife Dogari, Natalie Leonard, Kathryn Ward, Xiaoya Geng, Medha Raju, Jess Francis-Levin, Richard Gonzalez

**Affiliations:** 1 Institute for Social Research, University of Michigan, Ann Arbor, MI, USA; 2 Taubman College of Architecture and Urban Planning, University of Michigan, Ann Arbor, MI, USA; 3 School of Social Work, University of Michigan, Ann Arbor, MI, USA; 4 Anderson School of Management, University of California, Los Angeles, CA, USA

**Keywords:** Feedback, system design, research participants, engagement, qualitative

## Abstract

**Introduction:**

Declining participant engagement threatens human subjects research. Participant feedback systems (PFS) may combat this decline by empowering participants to evaluate their research experiences and share that feedback with researchers to identify targets for improvement. PFS signal that participant experiences are prioritized, making the request for feedback itself an intervention. PFS design work remains largely confined to clinical research. This exploratory study investigates the design parameters of extending PFS to nonclinical research. We conducted focus groups with nonclinical stakeholders: Experienced research participants (ERP) and research team members (RTM).

**Methods:**

ERP focus groups were organized by affinity (LGBTQIA+, BIPOC, persons with disabilities, neurodivergent, and a general group). RTM focus groups were organized by unit within the University of Michigan. Transcripts were analyzed using inductive thematic analysis.

**Results:**

Ten focus groups (ERP: 5, *n* = 25; RTM: 5, *n* = 26) identified key PFS design considerations: (1) motivations for feedback, (2) feedback collection, and (3) feedback delivery. ERP and RTM collectively preferred anonymous web-based surveys with six potential topic areas: communication, respect, being valued, receiving value, burden, and safety. Feedback delivery faced two key design tensions: balancing institutional standardization with study-specific insights and aligning leadership’s preference for high-level summaries with frontline staff’s need for detailed, real-time feedback.

**Conclusion:**

Expanding PFS to nonclinical research requires balancing centralization and study-specific flexibility. While centralization enhances consistency, the diversity of nonclinical studies necessitates adaptable implementation. A hybrid model is proposed to optimize feasibility. Future research should refine and test this model.

## Introduction

Contemporary research ethics recognizes research participants as partners in knowledge creation [1]. This view positions engagement in research not as a one-time transaction, but as a potentially sustained and evolving partnership. Participant research engagement operates across both *within-study domains* (e.g., co-design [2], enrollment, main study activities, study exit) and *between-study domains* (e.g., evaluating new enrollment opportunities, maintaining contact). Framing participant research engagement as a long-term behavior (analogous to civic participation, health behaviors, or consumer behaviors) invites insights from broader theoretical frameworks.

Promoting sustained participant engagement is essential to the research enterprise, yet evidence suggests participant engagement is waning across disciplines. Although comprehensive data covering all forms of engagement are limited, two critical indicators show significant strain: recruitment and retention. Over 76% of clinical trials experience delays or discontinuation due to poor enrollment [3,4]. Outside clinical trials, robust case tracking in national surveys reveals similarly troubling trends [5–7]. The Behavioral Risk Factor Surveillance System response rate fell from 71.4% in 1993 [8] to 44.6% in 2023 [9], the Health and Retirement Study declined from 81.6% in 1992 to 73.9% in 2020,[10] and the General Social Survey dropped from 82.4% in 1993 to 59.5% in 2018 [11]. These declines persist despite well-funded mitigation efforts [12–16], and they appear to be compounded by declining public confidence in science and scientists [17]. Together, these indicators point to deeper relationship problems between researchers and the broader public.

Leeper offers a compelling interpretation of this erosion of engagement as a common pool resource problem [18]. Akin to overfishing a pond, when researchers recruit without regard for collective impact, they exhaust the pool of willing participants. Stewardship of this resource is necessary. Yet this metaphor has limits. People are not passive resources to be harvested. Reframing participants as active contributors shifts the focus. Knowledge itself becomes the shared resource, and scientific research is a form of collective action that produces this public good [19]. Participants contribute as “experts by experience” and researchers as “experts by training”[20]. Both framings underscore a common point: the sustainability of research engagement hinges on the quality of researcher–participant interactions.

Growing evidence supports this view. Participants’ past research experiences can shape their future engagement decisions, influencing both individual choices and broader community trust [21–23]. Yet most research teams have few mechanisms for learning from participant experiences. The absence of structured feedback channels not only limits opportunities for improvement but may also reinforce the very disengagement that institutions hope to address.

Participant Feedback Systems (PFS) offer a promising solution. PFS are structured mechanisms that allow participants to evaluate their research experiences and share that feedback with researchers and institutions. Feedback may inform a range of improvements, from staff training and protocol refinement to institutional policy decisions. Moreover, research on consumer behavior suggests that the very act of requesting or providing feedback can positively affect future engagement, known as the “mere solicitation” and “mere measurement” effects [24].

The clinical research field has led the way in developing PFS, most notably through Rhonda Kost’s pioneering *Empowering the Participant Voice* (EPV) project. EPV has improved participant satisfaction, enrollment, and response rates across several institutions [25]. Yet despite these promising developments in clinical trials, PFS have not meaningfully extended into nonclinical human subjects research – but why not?

Part of the challenge is conceptual. Clinical research benefits from the alignment between “patient” and “participant” roles, making it easier to adapt preexisting healthcare feedback models. In contrast, social scientists interact with feedback systems that engage different audiences, such as teaching evaluations, tenure reviews, and employee performance evaluations. These forms of feedback engender less intuitive mental “stepping stones” for PFS development. Structural diversity is another barrier. EPV succeeds in part because clinical trials share common rhythms that support standardization. Nonclinical research spans a broader range of methodologies, populations, and procedures, complicating efforts to design one-size-fits-all tools.

To address this gap, we conducted an exploratory study to inform a PFS model for nonclinical human subjects research. Drawing on Kost et al.’s stakeholder-driven approach, we conducted focus groups with two stakeholder groups: experienced research participants (ERPs) from nonclinical studies and nonclinical research team members (RTMs). We used both direct inquiry (asking attendees about preferences for feedback design) and indirect inquiry (inviting them to describe their own positive and negative research experiences). This indirect method proved especially valuable in ERP groups: abstract questions about feedback often yielded limited responses, while personal stories surfaced implicit preferences and rich contextual insights. Together, these approaches allowed us to identify core content domains for participant feedback and clarify practical PFS design tensions. The discussion section proposes an initial framework for extending PFS beyond clinical trials.

## Materials and methods

### Study design and recruitment

We conducted separate focus groups with two stakeholder groups, ERP and RTM. The University of Michigan (U-M) IRB-HSBS determined that the study was exempt from ongoing oversight (HUM00247823). All attendees received an Information Sheet aligned with standard informed consent.

A participant-centered approach guided session design, similar in spirit to patient-centered approaches [26]. Groups were held in person or remotely via Zoom; hybrid sessions included technical support and assistant moderators. Recruitment materials described PFS only in vague terms to avoid biasing the discussion. The moderators began each session with this framing: “The goal of the [PFS] is to improve the research experiences of all individuals and communities, to build trust, and create pathways for communicating with research teams.”

Attendees engaged in three moderator-led activities: (1) stakeholder mapping to identify those with a vested interest in PFS, (2) research experience journey mapping activities, and (3) open-ended discussion of opportunities to provide/gather feedback, good/bad research experiences (ERP only), topic areas and use of feedback, and feedback delivery structure (RTM only). Pretesting showed that asking directly what a PFS should include often yielded limited responses. In contrast, prompting reflection on personal experiences produced richer input, and thematic analysis of those reflections revealed relevant content areas for feedback instruments. This indirect approach was used in the final protocol. Journey maps were constructed as multi-layered visual aids (see Fig S1), annotated and referenced during discussion to ground input in real-world experiences. Whiteboards (digital or physical) were used to organize ideas.

Sessions were audio and video recorded for transcription. Attendees also completed a brief online follow-up survey (≈ 5 minutes) to report their demographics and provide additional insights (Table S2). Differences between ERP and RTM focus groups are addressed next.

#### Experienced research participants (ERP)

ERP focus groups were conducted March-July, 2024 (average duration: 1.5 hours; compensation: $50). ERP were recruited through the U-M Health Research Volunteer Portal [27] and an additional U-M participant registry, using purposive sampling to amplify historically marginalized voices. Eligibility criteria included: (1) age ≥ 18, (2) comfort communicating in written and spoken English, (3) lived within a drivable distance to Ann Arbor, MI, and (4) enrollment in a nonclinical U-M research study within the past 12 months.

Sessions were organized into affinity groups (AG) to amplify underrepresented voices: [1] LGBTQIA + community, [2] Black, indigenous, and persons of color (BIPOC), [3] persons with disabilities, [4] neurodivergent community, and [5] a general group open to all. To account for intersectionality, participants self-selected into focus groups based on identity and scheduling availability. AGs provided space to raise identity-specific concerns; the general group allowed for cross-cutting input. Future research could incorporate additional perspectives, such as those of caregivers and rural residents.

#### Research team members (RTM)

RTM focus groups were conducted March-August, 2024 (average duration: 1 hour; compensation: $30). Eligible RTM (1) were aged 18 or older, (2) worked on a nonclinical human subjects research team at U-M (Ann Arbor), (3) currently interacted with research participants or expected to within six months, and (4) belonged to a team with at least two members.

Sessions were organized around five U-M research entities: the School of Social Work, the School of Public Health, the Ross Business School, the University of Michigan Transportation Research Institute, and the Institute for Social Research. These teams used diverse methodologies (quantitative, qualitative, and mixed) and engaged a wide range of populations in both experimental and observational studies. Each enrolled team identified up to six members for the focus group, aiming to include a range of research roles, from principal investigators (PIs) to undergraduate research assistants.

### Analysis

Transcripts were analyzed using inductive thematic analysis [28]. ERP and RTM perspectives were compared to examine how lived experience (ERP) and research role (RTM) shaped design tensions. Recurring themes were iteratively refined; illustrative quotes appear in the results. ERP quotes include demographic context (e.g., AG membership), while RTM quotes only specify team roles to minimize reidentification risk in a small population.

## Results

Findings are presented in four sections: (1) attendee demographics; (2) existing feedback practices and motivations; (3) feedback collection from research participants; and (4) feedback delivery to research teams. Existing practices and motivations are included as contextualizing factors to establish a baseline and clarify what each stakeholder group hopes to gain from the feedback process. Feedback collection (including content domains) and delivery are addressed because they represent core design decisions in building a PFS for nonclinical research.

### Demographics

Ten focus groups were conducted, five for RTM (*n* = 26) and five for ERP (*n* = 25). Demographics are summarized in Table S3. While modest in size, the samples likely reached thematic saturation [29]. However, see *Limitations and Future Directions* for a discussion of how to expand on this work.

Research teams varied widely in composition. RTM self-identified one or more team roles, with 9 (39%) reporting as PIs, 8 (35%) as Research Assistants or Associates, 2 (8.7%) as Participant Coordinators (PC), and 2 (8.7%) as Data Managers or Analysts. Additional roles accounted for approximately 22% of responses, including Lab Managers (LM), Lab Directors (LD), Project Managers/Coordinators (PM), Technicians, Interviewers, and Research Leads.

ERP session attendance was as follows: LGBTQIA+ (*n* = 3), BIPOC (*n* = 6), Persons with Disabilities (*n* = 6), Neurodivergent (*n* = 5), and a general group (*n* = 5). Due to the intersectionality, the number of participants in each focus group does not directly reflect total AG representation (Table S3). For example, LGBTQIA + group attendance was small, six ERP identified as LGBTQIA+. Quotes are tagged by self-identified AG, not focus group assignment.

### Contextual factors–existing feedback practices and motivations

Formal feedback practices were largely absent. Only one research team reported using a structured PFS, typically a single open-ended item appended to a research survey. RTM cited time constraints and limited technical support as barriers to implementation. ERP also reported a near-total absence of formal feedback opportunities. None reported encountering formal PFS, though some had offered unsolicited input to RTM. Instead, some described sharing their opinions through personal networks, affecting broader perceptions of research:

*No one really asked for feedback, so.* [long pause] *And I may have been informally in my social circle coming home saying “this study was good” and “this study was less than quality.”*

*- 68, Woman, White; AG: None Identified*

Three primary motivations for providing/soliciting feedback emerged from thematic analysis: (A) Refining Protocols and Training to Enhance Participant Experience, (B) Increasing Repeat Participation and Reducing Attrition, and (C) Producing Feasibility Evidence (Table 1). Understandably, (B) and (C) were only motivators to RTM.

**Table 1.** Motivations for gathering/providing feedback – themes and exemplars

Theme	Exemplar quotes
(A) Refining protocols and training to enhance participant experience	**RTM** *Quote 1:* *PC:* “[…] So I think probably what we would be interested is more the research process [RTM murmurs of agreement]. Like, yeah-” [Overlapping speech with PI] *PI:* [Overlapping speech with PC] “ – that kind of thing, like. What, what would have improved [participants’] experience. Like all the way from the recruitment flyer, or something that they saw or, um, [overlapping speech with PC] their-” *PC:* [overlapping speech with PI, completing PI’s sentence] “-their experience there in the session. And, and I think it’s also a really nice opportunity […] what kinds of things could we change to make it more comfortable or a better experience for them. Just to kinda get that, um, could be a good opportunity for that [unintelligible word].” *Quote 2:* *PI:* “If there’s an issue, I need to know that. [long pause] That to me this type of feedback system is very helpful because it could potentially be a way to visualize to [our large number of interviewers] who are literally going to be spread all over the United States, how they’re doing relative. […conversation continues…15 minutes later into discussion…] I’d want real time, like, very responsive feedback that I could give to the people in the field during fieldwork about, here’s something that really should have, here was a way to phrase this thing that really seemed to resonate with the people [participants] just like, this one interviewer has kind of got, got some skills here that, that’s resonating. Let’s, let’s take that into how we’re doing business.” **ERP** *Quote 1:* *Woman, 58, African American; AG: BIPOC:* “If I thought that my feedback was being used–like if there was some way for them to acknowledge that, like, in the past we have tweaked this study based on people’s feedback…then you feel like it’s going somewhere.” *Quote 2:* *Woman, 65, White; AG: Persons with disabilities:* “I would envision the research teams using participant feedback to guide them in how to interact with future participants, or even guide them in the construction of the study or the survey. That would make me more likely to provide feedback.” *Quote 3:* [The following two quotes are part of a single speech exchange] *Woman, 34, White; AG: None Selected:* “Make it better for future participants. I’ve done my part. Can’t change what happened for me. But like, let’s make the experience better for those that are going after me. [overlapping speech]” *Man, 76, White; AG: None Selected:* “[overlapping speech] Training. Universal training. There are research coordinators that go into the job that have no idea what they have to do, at least initially. Part of the–principal investigator gets a hold of that person and says, ‘Okay, you know, here’s a couple of calls, phone calls you can go to [pause] that are really, uh, challenged.’”
(B) Increasing repeat participation and reducing attrition	**RTM** *Quote 1:* *PI-1:* “…Maybe we need to get feedback there so that we can improve on the recruiting end…But the problem is that how could we entice them to come back again, right? …The excitement, you know, fades away.” […additional discussion] *PI-2:*“Like we’ve tried to give more money […] but money does not always seem to be the right solution.” *Quote 2:* “…this comes back to the participant, their experience, because what I want to make sure what we do is…reestablish a meaningful relationship with these folks [participants]…” *- PI; on recontacting after a gap in participation*
(C) Producing feasibility evidence	**RTM** *Quote 1:* *PI:* “I think the big thing that NIH worries about is burden, and every time I write [a grant] they’re like, ‘This is too much for [participants] to do.’ And yet they have really high response rates…” [overlapping speech] *PM:* “…So you can tell the NIH directly, this is what [participants] said! Not just, ‘we have 85% [adherence]’ but [participants] told us they were fine.”

Abbreviations - AG: Affinity Group; BIPOC: Black, Indigenous, Persons of Color; ERP: Experienced Research Participants; RTM: Research Team Members; PC: Participant Coordinator; PI: Principle Investigator; PM: Project Manager.

The introductory framing used in the focus groups (“to improve experiences, build trust, and create communication pathways”) may have influenced how some attendees articulated their motivations. Attendees expanded on this framework, offering examples of what PFS could accomplish. For example, both groups linked feedback to the goal of refining protocols and training to improve participant experiences (Table 1, Theme A). ERP also emphasized that improvements should be transparently shared with participants to demonstrate that their input was valued and acted upon (e.g., Table 1, Theme A, ERP Quote 1).

### Feedback collection from participants

#### Preferred form

Few ERP felt comfortable giving feedback directly to RTM; most preferred anonymous methods to encourage candid responses, especially web-based surveys, with some interest in SMS surveys. RTM ideated strategies for distributing survey links, including automatic redirection after a main research web survey, links in thank you emails, or QR codes printed on debriefing handouts, displayed on TV screens at community meetings, or sent via postal mail. They noted that accessibility and participant preferences would likely vary across different populations and research contexts.

#### Core content areas

This section outlines core content areas identified for inclusion in a feedback survey. As described previously, we used both direct and indirect inquiry to explore feedback content: attendees were asked about their preferred survey topics, and ERP were invited to reflect on their positive and negative research experiences. The indirect approach proved especially productive; most input that shaped these content areas came from spontaneous reflections during journey mapping and related discussion (Fig. S1; Table S2).

Six key content areas emerged: (A) Communication, (B) Respect, (C) Burden, (D) Feeling Valued, (E) Receiving Value, and (F) Safety and Security (Table 2). Exemplar quotes in Table 2 are drawn exclusively from ERP to center participant perspectives. While RTM also contributed to theme development, their sessions generated many protocol-specific content areas for feedback that were not broadly generalizable, such as questions about satisfaction with waiting rooms, whether training on study-specific technology should occur in person vs. self-directed, the learning value of participation for student subject pools, or, in the following example, a question about who is providing relief to a caregiver so that the caregiver can attend a research appointment:

**Table 2.** Core survey content areas – themes and exemplars

Theme	Exemplar quotes from experienced research participants
(A) Communication	*Quote 1:* *Man, 23, Hispanic or Latino; AG: BIPOC, LGBTQ+:* “I think every step of the way in the research studies I was able to participate in, they would always explain what was about to happen, how long it would take, and the types of questions they’d ask me. They kind of gave me a summary of every step, and that would help me prepare for what I’m going into.” *Quote 2:* *Woman, 25, White; AG: Neurodivergent:* “A bad research experience is definitely lacking of communication for any reason. Feeling rushed through anything… I had a question to that man who gave me the consent form, and he did not know how to answer it. And I was like, ‘Like, normally, you should know the answer!’” *Quote 3:* *Woman, 50, Middle Eastern; AG: BIPOC:* “I would actually read the report like if they sent it to me. But most times they do not send you the final product of the report. But you would like it.”
(B) Respect	*Quote 1:* *Woman, 59, White; AG: Persons with Disabilities:* “I think for me a good research experience is being respected, also. You know? It’s the ‘please,’ the “thank you,” the follow up. …And I’ve heard a lot of people saying the researcher met him at the door, but I haven’t heard a lot of people saying that, you know, “thank you.” *Quote 2:* *Man, 23, Hispanic or Latino; AG: BIPOC, LGBTQ+:* “Possible research groups say they’re looking for people whose gender is male … And so once I get the call saying …‘What sex were you assigned at birth?’ and I have to say female, they’re like, “Oh, …you do not actually qualify….”That has always been an issue…it’s like they’re afraid to say sex.” *Quote 3:* *Woman, 32, White; AG: LGBTQ+, Neurodivergent:* “There are a lot of people who aren’t very disability-informed, who maybe aren’t very neurodivergent-informed, all sorts of stuff. And I’ve just had experiences where it, like, takes me longer to do some things, like, especially, like writing. My hands are bad, and researchers are not always the best about it … [researchers are] just trying to get people out of there.”
(C) Being valued	*Quote 1:* *Woman, 58, African American; AG: BIPOC:* “[The researchers’] responses seem like they value what you’re saying. They respond in a way that makes it feel like […] you’re having a conversation and not like ‘I’m checking off a list of things that I need to get done’ … So I think that makes a difference too, at least for me.” *Woman, 33, African American; AG: BIPOC:* “And then there are some studies that actually will send me […] a little thank you message, email, like, ‘…we also appreciate you participating in the study and what you did, like, might have helped save someone’ or, you know, “helped with this new technology work for more people” or like “sales up” or something?”
(D) Receiving value	*Quote 1:* *Male, 49, White; AG: Neurodivergent:* “And also, to be honest, I mean, I probably wouldn’t participate in many studies if there was no compensation, you know, just to be transparent.” *Quote 2:* *Male, 49, White; AG: Neurodivergent:* “Also [I participate] for the social interaction, and just meeting different people and staying current.” *Quote 3:* *Woman, 33, African American; AG: BIPOC:* “I’ve been doing this for ten years and I really enjoy, like, helping and also being a part of, like, seeing, like, different technologies.” *Quote 4:* *Woman, 34, African American; AG: BIPOC*“I just know there’s a lot of less research on People of Color to back the science. So for that [I participate in] research.”
(E) Burden	*Quote 1:* *Woman, 34, White; AG: None Selected:* “Timing–respecting my timing. ‘You said that [this study] would take an hour and a half. I’ve been here for three hours!’” *Quote 2:* *Woman, 58, African American; AG: BIPOC:* “I would’ve been like, ‘…Why are you asking the same thing over and over again each day? Like, I would’ve given that feedback, but they didn’t give the opportunity.’
(F) Safety & security	*Quote 1:* *Woman, 50, Hispanic or Latino, African American; AG: BIPOC:* “I understand the need for demographics. At the same time, that it’s for the common good. […] Like ‘for the good of something’ is fantastic, but that’s not always the case. I do not know how to decipher that sometimes. And if you sell that information somewhere else, like, then what happens? You know? It’s like, how do you get control three hands down, you know?”

Abbreviations - AG: Affinity Group; BIPOC: Black, Indigenous, Persons of Color; LGBTQ+: Lesbian, Gay, Bisexual, Transgender, Queer, and other identities.

*PI-1: A consideration is caregivers are busy giving care. So, and can they leave their care partner for two hours, for like to get to [redacted: the study site] to do an in-person session? […] [overlapping speech]*-

*PI-2 (to PI-1): -[overlapping speech] I don’t know if you might find it useful to know from your [PI-1’s] participants, who’s taking care of the person that they’re used to taking care of while they’re in your study?*

The need to accommodate such hyper-specificity through customization is addressed later.

##### Content theme A – communication

The theme “communication” (A) encompasses the quality and transparency of communication between participants and research teams throughout the research process. Attendees noted that the communication dynamics significantly influenced the overall research experience. RTM highlighted recurring mismatches between participants’ expectations and actual experiences. ERP similarly reported that good communication helped them feel informed and prepared, whereas poor communication left them feeling unsettled and disengaged.

##### Content theme B – respect

The “respect” (B) theme refers to the recognition and consideration of participants’ identities, needs, and contributions throughout the research process. ERP desired respect to be shown through both words and deeds. Expressions of gratitude were important for individuals whose participation was affected by chronic illness or disability. Respect also included acknowledging personal information shared beforehand (e.g., body size) and being prepared to recognize and accommodate diverse body types, gender identities, neurotypes, and disabilities.

##### Content theme C – being valued

The “being valued” (C) theme refers to participants feeling that their individual contributions are recognized as meaningful and not interchangeable or perfunctory. This theme connects to communication and respect, but emphasizes reciprocity, making participants feel like active partners in discovery rather than passive subjects. For example, ERP felt valued when RTM practiced active listening and connected their individual contributions to broader societal impacts.

##### Content theme D – receiving value

The “receiving value” (D) theme refers to participants feeling that they gain something worthwhile from their involvement in research. While financial incentives were a key extrinsic motivator, ERP emphasized that value extends well beyond payment. Compensation was seen as a necessary baseline, necessary but not sufficient for a positive experience. ERP also identified intrinsic motivations for participating in research, including social engagement, contributing to a greater purpose, and satisfying curiosity. Understanding the full spectrum of value participants seek, both extrinsic and intrinsic, is necessary to capture whether research teams are delivering this value.

##### Content theme E – burden

The “burden” (E) theme relates to the resource strain experienced by participants during the research process, whether physical, emotional, cognitive, or financial. ERP experienced overburden in various ways, often arising from boundaries being overstepped or unmet expectations regarding effort, discomfort, and time commitments. The repetitive nature of study tasks also contributed to fatigue and disengagement. Perceptions of burden were highly individual, and participants noted that accommodations from researchers could alleviate these challenges. Clear communication of needs and expectations helped reduce participants’ burden and demonstrated respect for their time.

##### Content theme F – safety and security

The “safety & security” (F) theme encompasses participants’ sense of physical, psychological, and informational protection throughout the research process. Data security concerns were prominent. ERP expressed significant worry about the potential misuse or breach of their personal and demographic information, including fears about data being “sold” or shared without consent. Although consent forms attempt to address such concerns, feedback surveys could assess the effectiveness of these trust-building measures.

#### Other considerations for feedback collection

Other key design elements of the feedback survey included transparency, autonomy, the balance between open-ended and closed-ended questions, compensation, and the inclusion of vulnerable populations.

##### Transparency and autonomy

ERP wanted transparency regarding who would receive their feedback and the purpose of the information collected. To tailor their feedback, ERP emphasized the need to know whether responses would be shared only with researchers they interacted with or distributed more widely. ERP also emphasized the importance of understanding why certain questions (particularly demographic ones) were included and how their answers would be utilized. ERP were mistrustful when a question’s relevance was unclear, particularly regarding sensitive topics like income. The inclusion of demographic questions in a feedback survey was also controversial among RTM; some viewed them as unnecessary, while others considered them essential for identifying disparities in research experiences.

Even when information-gathering intent was clear, ERP valued their autonomy to skip questions. When clear options to bypass sensitive questions were unavailable, some ERP abandoned surveys entirely. This created frustration, as ERP believed abandonment meant their partial responses would not be used.

##### Balance of open-ended vs. closed-ended questions

The balance between open-ended and closed-ended questions sparked mixed reactions among both RTM and ERP. Some ERP found excessive open-ended questions burdensome, while others vastly preferred them, feeling that scale-based questions diminished the value of their feedback or made the process perfunctory. RTM reactions also varied, often splitting along leadership versus operational roles as discussed later in “Feedback Delivery to Researchers.”

##### Compensation for feedback

Both RTM and ERP expressed that compensation for feedback was ideal from the perspectives of fairness and honoring participants’ labor value. However, offering material incentives was controversial for several reasons. RTM’s concerns included compensation potentially biasing feedback, fostering transactional relationships, encouraging superficial responses, or creating inequities in underfunded research projects where teams might not be able to afford compensation. RTM also raised legal and logistical challenges; tracking and reporting participant feedback incentives for tax and accounting purposes could compromise the perceived confidentiality (see Discussion for proposed solutions).

##### Vulnerable populations

Two of the five participating research teams routinely worked with vulnerable populations: minors under the age of 18 and individuals with cognitive decline. These teams recognized the value of gathering feedback directly from these groups, but struggled with ethical and regulatory challenges. These challenges were compounded by standard consent procedures, such as requiring signatures from parents or legally authorized representatives, which conflicted with the goal of maintaining anonymous feedback. These examples underscore the need for deeper reflection on whose voices PFS may miss. While no solutions emerged from the focus groups, raising awareness is a crucial step in expanding access (e.g., caregiver proxies, prospective permission from caregivers to solicit feedback from minors, and capacity-adapted tools).

### Feedback delivery to researchers

Only RTM focus groups explored how participant feedback should be delivered to researchers. Preferences varied based on team roles, research methodologies, and participant populations.

#### Data access and format

RTMs had differing views on data access and reporting. While all desired some form of feedback summary, some also valued the ability to download raw data for independent analysis. Preferences differed regarding feedback types: staff with direct participant contact and supervisors found qualitative feedback more actionable, although they noted that such content could be overwhelming or conflicting without summarization. Other RTMs, especially those in leadership positions, preferred Likert scales for tracking trends. All agreed on the need for secure sign-on, update notifications, and role-based restrictions to protect both data and team members. There was sensitivity to the need for a moderator to ensure feedback was delivered constructively.

#### Delivery frequency and summary level

Preferences for feedback frequency and format varied by role. Most PIs preferred periodic, high-level summaries, whereas staff with day-to-day study responsibilities emphasized the need for immediacy and detail:

PI-1: *…It could be a monthly newsletter, I mean, that one’s the easiest one.*

PI-2: *Maybe quarterly, too. I feel like that’s how long a lot of our studies run.*

LD: *But if there’s something alarming, though, we want to know. I mean, if [participants] say something, and they’re assuming that we’re going to see that feedback [we don’t want to] see it in three months.*

#### Benchmarking, comparison, and data sharing

RTMs also desired both within-study and between-study comparisons to better interpret participant experiences, for example: comparing trends across demographic groups, monitoring changes over time, evaluating the impact of protocol improvements, and benchmarking against similar studies. Although these approaches imply some level of data sharing, necessary for benchmarking, RTMs expressed hesitation about feedback being used punitively by governing institutions, underscoring the need for safeguards.

## Discussion

This exploratory study advances the development of PFS for nonclinical human subjects research. We identified key content domains for feedback surveys that differ meaningfully from those established in clinical trial research [30]. Beyond content considerations, we identified two central design tensions: balancing feedback needs (1) across roles (leadership versus frontline staff) and (2) across organizational levels (centralized standardization versus decentralized customization). We propose a scalable PFS model and examine these tensions concerning its design.

### Operationalizing a scalable PFS

**Figure 1.**
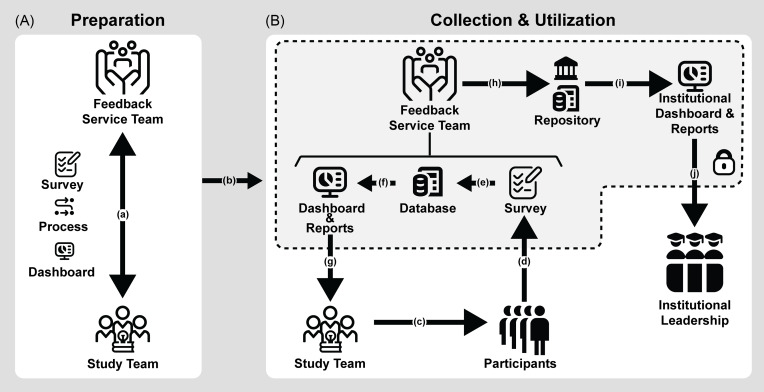
Feedback system design. The system operates in two phases: [A] preparation and [B] collection & utilization. During preparation, the feedback service team collaborates with the study team to (a) customize the survey, solicitation process, and dashboard. Once approved by the study team, feedback service team, and any involved ethical oversight bodies (e.g., IRB), the system can (b) advance to collection & utilization. In collection & utilization, the study team (c) distributes the survey to participants, who (d) submit anonymous feedback. This feedback is (e) securely stored in a database and (f) summarized in a dashboard. The study team (g) reviews the feedback to inform study improvements. To protect study team anonymity, the feedback service team (h) de-identifies data before adding it to an institutional repository. Finally, the repository data is (i) compiled into dashboards or reports for (j) Institutional Leadership. Symbols are original or modified from www.flaticon.com.

To address needs across organizational levels, Figure 1 presents a hybrid PFS model anchored by a centralized Feedback Service Team (FST), which lowers implementation barriers and acts as an intermediary among research teams, participants, and institutional leadership. The system operates in two phases. In the preparation phase, the FST collaborates with research teams to add study-specific content into a Custom Module of the feedback survey. Customization addresses RTMs’ desire for feedback on hyper-specific content areas that are unique to their study. In parallel, a Core Survey module of universal topic areas is maintained for uniformity and comparison across studies. Distribution methods should be adapted to the study population to maximize response rates. In the collection and utilization phase, anonymous participant feedback is gathered and summarized in automated dashboards to support study-level improvements while reducing the burden on research teams. De-identified data are compiled into reports for study leadership and aggregated across studies into an institutional repository, enabling both targeted insights and broader organizational learning.

This structured yet flexible model supports feedback use at both micro and macro levels but requires careful attention to logistical, economic, ethical, and technical challenges. For example, participant compensation for feedback merits consideration; while compensation for the labor of feedback is desirable, RTMs raised concerns that payment could bias feedback, foster transactional relationships, or create inequities in research teams unable to afford compensation for feedback. Tracking and reporting incentives for tax and accounting purposes could also compromise the perceived confidentiality of feedback. Unconditional prepayment (wherein all participants are compensated upfront for feedback, regardless of completion) is a well-established, cost-effective method for boosting response rates [31] that could enhance anonymity by breaking the link between feedback submission and payment. Alternative approaches such as third-party management systems or voucher/token systems could also further protect confidentiality. Yet these mechanisms do not address the underlying inequities in research team funding, a problem that remains to be solved at the institutional level.

### Specific recommendations for nonclinical feedback survey design

At the center of the proposed PFS is the feedback survey itself. The feedback survey should balance the informational needs of research teams against participant burden, autonomy, and trust. Our findings suggest the Core Survey module for a nonclinical feedback survey should center six content domains: Communication, Respect, Being Valued, Receiving Value, Burden, and Safety and Security. These domains appeared to be universal across the studied nonclinical research contexts. Within the Communication domain specifically, the feedback survey should assess general communication practices, rather than focusing on consent processes as clinical research feedback systems may. This broader view is necessary given that consent requirements and protocols vary substantially across the methodological spectrum of nonclinical research. Beyond content, nonclinical feedback survey design must also accommodate varying sensitivities among participants. To this end, the survey should provide a brief rationale to the participant for asking sensitive questions, and sensitive questions should be obviously skippable. Demographic questions, for instance, can be highly sensitive due to the potential for reidentification in smaller subpopulations. Offering both an explanation and an opt-out option for the demographic section in particular helps participants balance confidentiality concerns with the value of contributing to group representation (e.g., revealing disparities in research experiences). Similarly, open-ended questions should be easily bypassed given polarized opinions on their value versus participant burden. Collectively, these design choices signal that participant comfort and voice are prioritized alongside the needs of researchers and institutions.

### Feedback needs by role

Establishing a robust survey instrument is one challenge; the other is operationalizing the system to effectively support diverse users. Thus, the model must consider the varied feedback needs across roles within research teams, which impacts data delivery and frequency. RTM in leadership roles preferred infrequent, summarized feedback, while frontline staff favored more frequent, detailed input. These patterns align with organizational research showing that individuals in lower-hierarchical roles often seek detailed feedback to reduce uncertainty in the performance of duties, while those in leadership prefer aggregated reports for strategic planning [32]. This dynamic has implications for PFS design, including balancing closed-ended questions, which meet leadership needs for summary, with open-ended items that offer the nuanced detail valued by frontline staff, as well as tailoring feedback frequency to role.

PFS may also function as a form of performance evaluation for frontline staff, which raises important considerations. Feedback interventions can backfire, decreasing future performance if implemented without regard for framing, norms, and individual factors [33]. Raw feedback may be unhelpful, or even harmful, without appropriate interpretation. Additionally, anonymous feedback may not be assignable to a specific RTM. If feedback can be individualized, a moderating layer is essential. While an FST can offer support, they lack insight into team dynamics and individual context. Supervisors are best positioned to contextualize PFS data, but nonclinical teams may lack this structure. Whether and how PFS should inform individual performance evaluation remains an open question and warrants further study.

### Feedback needs by organizational level

Existing PFS models in clinical research demonstrate clear advantages of centralized systems and have been effective in improving participant experience and engagement [34,35]. However, as described in the Introduction, extending centralized PFS to nonclinical research presents challenges due to greater methodological heterogeneity, fewer regulatory requirements, and more diverse team structures. Our hybrid model offers one potential solution, but concerns remain. Informal conversations with social science colleagues revealed apprehension about institutional overreach and the possible misuse of centralized PFS data in high-stakes contexts, such as tenure reviews. Although these concerns were not raised in formal interviews, they reflect broader cultural dynamics that need to be addressed. The success of PFS depends not only on technical soundness but also on trust between all parties.

Importantly, although centralization can reduce barriers by providing resources, it is not essential for success. Decentralized PFS approaches, supported by shared templates and communities of practice, offer a viable alternative that may better align with broader academic values and culture. Ultimately, we must weigh the trade-offs between infrastructure and autonomy in designing PFS that are both effective and acceptable.

### Limitations and future directions

This study was conducted at a single Midwestern R1 institution, which limits its generalizability. While focus groups were small and not nationally representative, the goal of this exploratory qualitative phase was transferability, not representativeness [36]. Larger samples, multiple institutions, and a wider range of study types are needed to assess cross-context applicability.

Self-selection bias may also have influenced findings, as more engaged participants and research teams receptive to feedback were more likely to participate. Moreover, PFS cannot address challenges at earlier stages of the research lifecycle (e.g., recruitment), where survivor bias remains a concern. Strategies are needed to reach nonparticipants and never-participants.

The next steps in this research program include gathering input from stakeholders to refine the PFS model, prototyping individual PFS components, and evaluating design trade-offs (e.g., hesitancy regarding demographic questions and role-based feedback needs). Success metrics should be developed at both the institutional and team levels.

## Supporting information

10.1017/cts.2025.10184.sm001 Carmichael et al. supplementary material Carmichael et al. supplementary material

## Supplementary material

The supplementary material for this article can be found at https://doi.org/10.1017/cts.2025.10184.
