# Supplementary material for: Extending participant feedback beyond clinical studies: A modular system designed to connect researchers and participants
Source: J Clin Transl Sci. 2025 Nov 3;9(1):e258. doi: 10.1017/cts.2025.10184 (PMC12766513; doi:10.1017/cts.2025.10184)
Supplement: Carmichael et al. supplementary material [file S2059866125101842sup001.docx]

# SUPPLEMENTARY MATERIAL


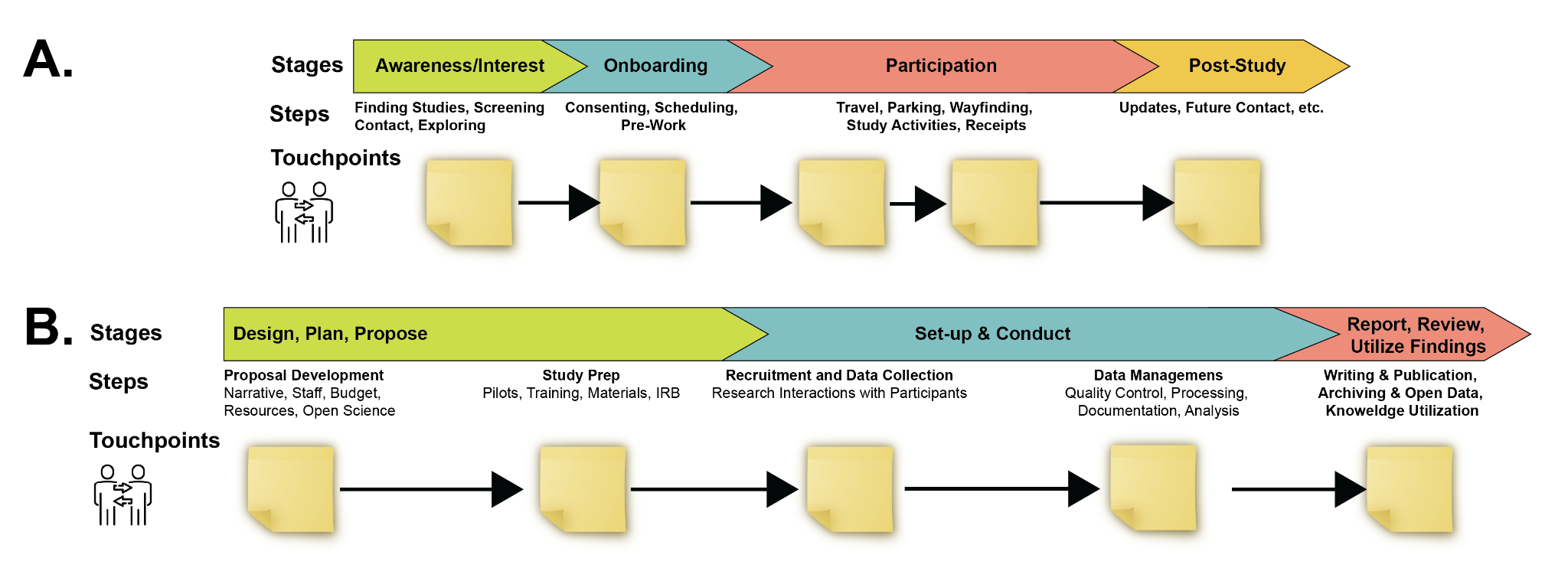


**Fig. S1. Journey Mapping Focus Group Activity Guide.** The journey map is a visual aid used to ground the Research Team Members (RTM) and Experienced Research Participants (ERP) in their real-world experiences as agents in the research process. Through layers of annotation and revision, the focus group attendees co-constructed visual representations of their ‘journey’ through research with a moderator’s aid. Initial structures were provided to open conversation (chevrons labeled to mark broad Stages and Steps). These differed for (A) ERP and (B) RTM and could be revised by the attendees as needed. Attendees outlined the steps in their journey via digital or physical sticky notes. Touchpoints (interactions) between researchers/participants were then marked using colored dots, stars, and other symbols as desired by attendees. Journey maps were displayed throughout the remainder of the focus group to serve as scaffolding when identifying areas of existing friction (difficulties, areas of improvement, etc.), and opportunities to solicit/provide/utilize participant feedback (see Table S2). *Symbols are original or modified from www.flaticon.com.*

| **Table S2:** Focus Group Discussion Questions and Follow-Up Survey Question | |
| --- | --- |
| **Experienced Research Participants (ERP)** | **Research Team Members (RTM)** |
| *Opportunities to Provide Feedback*   1. Have you been asked to provide feedback to a research team before? 2. What kind of feedback did you provide? 3. Why did you provide feedback? 4. Are there right times to provide feedback? | *Opportunities to Gather Feedback*   1. What feedback mechanisms are currently in practice? 2. What expectations do you have for future feedback mechanisms? 3. How is feedback incorporated currently? 4. What future expectations do you have for incorporating feedback? |
| *Defining Good/Bad Research Experiences**   1. What does a ‘Good Research Experience’ mean to you? 2. What does a ‘Bad Research Experience” mean to you? 3. What would have made it better? | *Topic Areas and Use*   1. What type of topics should participants provide feedback on? 2. Why? |
| *Topic Areas and Use*   1. What do you wish research teams would ask about your research experiences? 2. What topic areas are “off limits” for a feedback survey? 3. How do you envision research teams using participant feedback? 4. What would make you more likely to provide feedback to a research team? 5. How long would you want the feedback survey to be? 6. How do you want to give feedback? [Example: SMS, Email, QR Code, etc] | *Feedback Delivery Structure*   1. What should the Participant Feedback System look like? [Example: Reports, CSV, Dashboard, etc – only provided if scaffolding needed] 2. Who will be the receiver of the system? [Individual] 3. In what form will it be received? [Process] 4. How Frequently? [Time] |
| An additional open-ended question was administered via an online follow-up survey to both RTM and ERP focus group attendees in order to capture additional insights:  *The time constraints of a focus group can make it difficult for everyone to have the opportunity to fully share their thoughts. Was there anything else you wanted to share about the development of a Participant Experience Feedback System at U-M? Use the space below to communicate your thoughts with the BRITE design team.* | |
| ***  The questions under Defining Research Experiences function as a form of indirect inquiry, helping to identify potential topic areas for inclusion in a Participant Feedback System. Because they follow the journey-mapping activity, attendees are primed with specific, concrete examples, making it easier to respond. The rationale is that definitions of “good” or “bad” experiences can identify highly salient domains of the research encounter that are likely to shape participants’ overall evaluations and, therefore, worth capturing in feedback instruments. | |

**Table S3.** Focus Group Demographics

| **Characteristic** | **Experienced Research Participant** **(ERP)** N = 25 | **Research Team Member** **(RTM)** N = 26 | **Overall**  N = 51 |
| --- | --- | --- | --- |
| Age |  |  |  |
| Mean (SD) | 48 (17) | 42 (14) | 45 (16) |
| Median | 47 | 41 | 43 |
| Min, Max | 23, 76 | 19, 72 | 19, 76 |
| (Missing) | 0 | 6 | 6 |
| Gender, n (%^a^) |  |  |  |
| Woman | 14 (56) | 24 (92) | 38 (75) |
| Man | 9 (36) | 1 (3.8) | 10 (20) |
| Another gender | 2 (8.0) | 0 (0) | 2 (3.9) |
| I don't know | 0 (0) | 1 (3.8) | 1 (2.0) |
| Race, n (%^a^) |  |  |  |
| White | 18 (78) | 16 (62) | 34 (69) |
| Asian | 1 (4.3) | 4 (15) | 5 (10) |
| Black | 4 (17) | 5 (19) | 9 (18) |
| Race Not Listed | 0 (0) | 1 (3.8) | 1 (2.0) |
| (Missing) | 2 | 0 | 2 |
| Ethnicity, n (%^a^) |  |  |  |
| Hispanic or Latino | 3 (12) | 1 (3.8) | 4 (7.8) |
| Not Hispanic or Latino | 22 (88) | 25 (96) | 47 (92) |
| Education, n (%^a^) |  |  |  |
| High school diploma or equivalent | 1 (4.0) | 0 (0) | 1 (2.1) |
| Some college, but did not graduate | 4 (16) | 2 (8.7) | 6 (13) |
| Associate or 2-year college degree | 2 (8.0) | 0 (0) | 2 (4.2) |
| Bachelor’s or 4-year college degree | 7 (28) | 6 (26) | 13 (27) |
| Master’s degree | 8 (32) | 5 (22) | 13 (27) |
| Doctoral degree, professional degree or above | 3 (12) | 10 (43) | 13 (27) |
| Prefer not to respond | 0 (0) | 0 (0) | 0 (0) |
| (Missing) | 0 | 3 | 3 |
| Income, n (%^a^) |  |  |  |
| ≤ $24,999 | 5 (20) | 0 (0) | 5 (10.4) |
| $25,000-$49,999 | 1 (4.0) | 3 (13) | 4 (8.3) |
| $50,000-$99,999 | 9 (36) | 4 (17.4) | 13 (27.1) |
| ≥ $100,000 | 6 (24) | 13 (56.5) | 19 (39.6) |
| Does Not Know | 0 (0) | 1 (4.3) | 1 (2.1) |
| Prefer not to respond | 4 (16) | 2 (8.7) | 6 (12.5) |
| (Missing) | 0 | 3 | 3 |
| Identifies as LGBTQ+, n (%^a^) |  |  |  |
| No | 17 (68) | 13 (57) | 30(63) |
| Yes | 6 (24) | 6 (26) | 12 (25) |
| Does Not Know | 0 (0) | 2 (8.7) | 2 (4.2) |
| Prefers Not to Respond | 2 (8.0) | 2 (8.7) | 4 (8.3) |
| (Missing) | 0(0) | 3 | 3 |
| Identifies as POC or BIPOC, (%^a^) |  |  |  |
| No | 17 (68) | 14(61) | 31 (65) |
| Yes | 7 (28) | 7 (30) | 14 (29) |
| Does Not Know | 1 (4.0) | 2 (8.7) | 3(6.3) |
| (Missing) | 0 | 3 | 3 |
| Expressed Affinity for Neurodivergent Community, n (%)^ab^ |  |  |  |
| No | 7 (28) | NA | 7(28) |
| Yes | 18 (72) | NA | 6 (24) |
| (Missing) | 0(0) | 26 | 26 |
| Expressed Affinity for Disability Community, n (%)^ab^ |  |  |  |
| No | 15 (60) | NA | 15(60) |
| Yes | 10 (40) | NA | 10 (24) |
| (Missing) | 0(0) | 26 | 26 |

Abbreviations - BIPOC: Black, Indigenous, Persons of Color; ERP: Experienced Research Participants; LGBTQ+: Lesbian, Gay, Bisexual, Transgender, Queer, and other identities; POC: Persons of Color; RTM: Research Team Members

^a^Percentages do not include missing data. Although all RTM were aged 18 or over, due to a programming error, the exact age question was not asked of two RTM. All other examples of missing data for RTM were due to intentional non-response. ^b^Question asked only of ERM, not RTM.
